# Comparison CT radiation dose parameters, DLP and CTDI between two prominent tertiary care hospitals in Delhi NCR

**DOI:** 10.6026/973206300214991

**Published:** 2025-12-15

**Authors:** Lalit Gupta, Ashok Sharma, Ashish Kumar Shukla, Sumit Tyagi

**Affiliations:** 1 Department of Radiodiagnosis and Imaging Technology, Santosh Deemed to be University, Ghaziabad, Uttar Pradesh, India

**Keywords:** CT radiation dose, DLP, CTDI, tertiary care hospitals, radiation safety

## Abstract

The variations in radiation exposure levels and dose management protocols across hospitals pose challenges in optimizing CT radiation doses. Therefore, is it of interest to compares the CT radiation dose parameters, including Dose Length Product (DLP) and Computed Tomography Dose Index (CTDI), across two prominent tertiary care hospitals in the Delhi NCR region. The research aims to evaluate differences in radiation exposure levels, highlighting the variations in dose management protocols. Data was collected from routine CT scans to determine the dose levels at each hospital. This study contributes to the understanding of radiation exposure management by comparing CT radiation dose parameters across two major hospitals in Delhi NCR, offering insights into dose optimization practices.

## Background:

The use of computed tomography (CT) imaging is increasing since last few decades due to its superior diagnostic capabilities and ability to provide rapid and detailed imaging of internal structures [1]. While CT scans are indispensable in modern medical diagnostics, they are also a significant source of ionizing radiation exposure to patients, increasing concerns about potential long-term health risks, such as radiation-induced malignancies [2]. So it is important to monitor and minimize radiation dose without compromising diagnostic quality in radiology [3]. Key metrics used to quantify radiation dose in CT imaging include the Dose Length Product (DLP) and the Computed Tomography Dose Index (CTDl) [4]. These parameters provide valuable insight into the radiation burden imparted to patients during CT scans and help establish benchmarks for dose optimization. DLP accounts for the total radiation dose over the length of the scanned region. At the same time, CTDI reflects the average dose delivered to a standardized phantom volume during a single axial scan. Both parameters are associated with assessing radiation exposure and ensuring compliance with international dose reference levels (DRLs) [5]. Therefore, it is of interest to report and compare these parameters across different healthcare settings to highlight potential variations and opportunities for improvement in radiation safety practices.

## Methods:

This study employed a cross-sectional observational design to systematically evaluate and compare the CT Dose Length Product (DLP) and Computed Tomography Dose Index (CTDI) in two tertiary hospitals within the Delhi National Capital Region (NCR). The cross-sectional approach facilitated the collection of data at a single point in time, offering a snapshot of prevailing conditions and practices. The study was conducted at the Department of Radiodiagnosis, Santosh Medical College and Hospital, Ghaziabad, and the Department of Radiodiagnosis and Interventional Radiology, AIIMS, New Delhi. These two institutions served as the primary locations for data collection and analysis regarding radiation exposure during CT scans. The study participant included patients undergoing CT scans at the two tertiary hospitals. Adult patients were included, while pediatric cases were excluded to maintain homogeneity in the sample. A total of 200 patients were included based on non-probable consecutive sampling technique considering inclusion and exclusion criteria for the duration of 6 months (June 2024 to Dec 2024). Data collection was conducted in collaboration with the radiology departments of the participating hospitals. CTDI and DLP values were extracted from CT scanner software logs and records. Information regarding the make and model of CT scanners was also collected to understand the influence of technological factors. Ethical approval was obtained from the respective institutional review boards (IRBs) of the participating hospitals. Patient confidentiality and data privacy were rigorously maintained throughout the study. The collected data were entered in Ms-excel and run in to IBM SPSS V.25. Statistical analyses, including descriptive statistics were performed to examine the correlation between DLP and CTDI across the two hospitals. Subgroup analyses were conducted to explore variations based on patient demographics, scan protocols, and scanner models. The comprehensive analysis aimed to elucidate the factors influencing CT radiation dose variations in the designated tertiary hospitals.

## Results:

Table 1 (see PDF) shows the demographic characteristics of the study participants from the Santosh Medical College and Hospital and AIIMS, New Delhi. An equal number of patients (n=100) were included from each hospital, making up a total of 200 adult participants. The mean age of patients at Santosh Medical was 45.2 years (±14.3), while at AIIMS it was slightly lower at 43.7 years (±15.1), resulting in an overall mean age of 44.5 years (±14.7) across both centers. In terms of gender distribution, Santosh Medical had 62 male and 38 female patients, while AIIMS had 59 male and 41 female participants. Overall study was conducted among 121 males and 79 females. As shown in Table 2 (see PDF), For Head CT scans, SMC reported a DLP range of 880-1065 mGy.cm and a CTDI range of 50-55 mGy, whereas AIIMS reported lower values, with a DLP range of 690-935 mGy.cm and CTDI range of 40-45 mGy. In Chest CT, SMC showed a narrower DLP range of 150-170 mGy.cm and CTDI of 5-6 mGy, compared to a wider DLP range of 130-190 mGy.cm and slightly lower CTDI of 4-6 mGy at AIIMS. For Abdomen CT, SMC reported higher dose values (DLP: 350-400 mGy.cm, CTDI: 8-10 mGy) than AIIMS (DLP: 250-450 mGy.cm, CTDI: 6-9 mGy), showing overall higher radiation output at SMC across most scan types. As seen in Table 3 (see PDF). Head CT scans showed significantly higher mean CTDIvol at SMC (53 ± 2 mGy) than at AIIMS (44 ± 2 mGy), with a p-value of <0.001, showing a highly significant difference. Similarly, for Chest CT, the mean CTDIvol was higher at SMC (5 ± 2 mGy) compared to AIIMS (4 ± 2 mGy), with a statistically significant p-value of 0.001. For Abdomen CT, the CTDIvol at SMC (7 ± 2 mGy) also exceeded that of AIIMS (6 ± 2 mGy) and this difference was statistically significant (p = 0.011). These findings reflect a consistently higher radiation output at SMC. As shown in Table 4 (see PDF). Head CT at SMC had a significantly higher mean DLP (972.5 ± 53.4 mGy.cm) than AIIMS (812.5 ± 70.7 mGy. cm), with a p-value of <0.001. Interestingly, for Chest CT, the mean DLP was identical at both centers (160.0 mGy.cm), and the difference was statistically non-significant (p = 0.996). For Abdomen CT, the mean DLP at SMC (375.0 ± 14.4 mGy.cm) was slightly higher than that at AIIMS (350.0 ± 57.7 mGy.cm), but this difference did not reach statistical significance (p = 0.080).

## Discussion:

Our study shows significant differences in CT radiation dose parameters CTDIvol and DLP-between Santosh Medical College (SMC) and AIIMS, New Delhi, using comparable patient populations and common scan types (Head, Chest, and Abdomen). The findings showed that mean CTDIvol and DLP values were consistently higher at SMC, particularly for Head CT (CTDIvol: 53 ± 2 vs. 44 ± 2 mGy, p < 0.001; DLP: 972.5 ± 53.4 vs. 812.5 ± 70.7 mGy.cm, p < 0.001), reflecting statistically significant differences in scanner output and protocol settings. These results are in line with the work of Smith-Bindman et al. (2009) [6], who conducted a large-scale analysis across multiple healthcare facilities and found that radiation doses for identical CT examinations varied by as much as tenfold, depending on institutional practices and protocols used. Similarly, Mettler *et al.* (2008) [7] documented wide variations in CT radiation doses among U.S. hospitals, showing that local factors such as scanner type, protocol standardization, and technologist expertise can influence patient exposure. International studies have also reported dose disparities between centers. A dose audit conducted in the UK by Shrimpton *et al.* (2011) [8] for the National Health Service showed that average CTDIvol values for Head CT ranged from 40 to 60 mGy, with DLP values between 750 and 1100 mGy.cm, values consistent with the range reported in our study [8]. In our findings, AIIMS showed relatively lower values, likely due to the use of a newer-generation scanner (SOMATOM Definition Flash), which includes advanced dose-reduction technologies such as automated exposure control (AEC) and iterative reconstruction techniques. A study by Kalra *et al.* (2004) [9] emphasized the importance of protocol optimization and use of low-dose technologies in reducing unnecessary radiation exposure, even in high-volume tertiary care settings. AIIMS's lower dose metrics, particularly for Head and Abdomen CT scans, may reflect more stringent implementation of such optimization strategies. On the contrary, slightly higher values at SMC may be attributed to older scanner models (SOMATOM GO), non-optimized protocols, or higher baseline tube current-time products used during scanning. Interestingly, the difference in Chest CT DLP values between the two centers was negligible (160.0 mGy.cm in both), with a p-value of 0.996. This might show that protocol standardization for thoracic imaging has been better established across institutions, a finding that aligns with the observations of Tsapaki *et al.* (2006) [10], who noted less inter-center variability in Chest CT doses compared to Head and Abdomen protocols across European centers. The implications of these findings are significant. Radiation exposure from CT is cumulative and is associated with long-term cancer risk, especially in high-risk populations [11]. Therefore, efforts to harmonize protocols and reduce unnecessary variability are vital. Our findings emphasize the need for ongoing audits, dose-tracking systems, and implementation of national and international Diagnostic Reference Levels (DRLs), as showed by the International Commission on Radiological Protection (ICRP) and the American College of Radiology [12].

## Conclusion:

This study compared CT radiation doses (CTDIvol and DLP) between Santosh Medical College and AIIMS. Results showed significantly higher CTDIvol and DLP values at Santosh Medical for head CT scans (p <0.001), with similar trends observed for chest and abdomen CTs, though the DLP difference in abdomen CT was not statistically significant. These differences may be due to variations in scanner models, protocols, and dose optimization practices. The findings show the need for standardized CT imaging protocols to minimize unnecessary radiation exposure and promote patient safety.
